# Enhanced Surgical Safety through Protective Penetrating Keratoplasty: A Retrospective Analysis of 22 Cases

**DOI:** 10.1155/2024/2718527

**Published:** 2024-04-05

**Authors:** Tian Yang, Miguel O. M. Castellanos

**Affiliations:** Department of Ophthalmology, Hermanos Ameijeiras Clinical Surgical Hospital, Postal Code: 10400, Havana, Cuba

## Abstract

**Aim:**

To assess the surgical safety, postoperative outcomes, and the impact of PPK on corneal endothelial cell density through a retrospective analysis of 22 cases. *Settings and Design*. A retrospective cross-sectional observational study was executed at Hermanos Ameijeiras Hospital from February 2018 to December 2021, involving 22 patients (22 eyes) who were unresponsive to other medical treatments and had a corrected distance visual acuity of ≤0.1.

**Methods and Materials:**

Patients underwent PPK, with surgical procedures and postoperative care documented. Statistical analysis was performed on qualitative and quantitative variables to evaluate the surgical outcomes and the corneal endothelial cell density changes postoperation.

**Results:**

All surgeries demonstrated a significant improvement in postoperative visual acuity (*p* ≤ 0.001) and recorded a 9.2% decrease in the corneal endothelial cell density at 12 months. Noteworthy complications included one case of intraoperatively discovered haptic dislocation and one postoperative bacterial keratitis.

**Conclusions:**

PPK could potentially mitigate perioperative complications, ensure graft clarity, and reduce corneal endothelial cell loss, presenting itself as a viable alternative to traditional PK. Although the results are encouraging, larger-scale studies are essential to validate the benefits and applicability of PPK in broader clinical settings.

## 1. Introduction

In the realm of corneal transplantation, recent advancements have prominently positioned endothelial keratoplasty (EK) and deep anterior lamellar keratoplasty (DALK) as the preferred techniques for specific corneal pathologies. EK has been transformative in managing endothelial dysfunctions, while DALK is chiefly employed in keratoconus cases [1, 2]. Despite these innovations, the practical application of these techniques varies, influenced by the steep learning curve of EK and its substantial equipment requirements, especially pronounced in resource-constrained settings of developing countries [3, 4]. The Eye Bank Association of America reports contrasting procedural numbers for postcataract surgery edema, with penetrating keratoplasty (PK) still in considerable use alongside EK, both internationally and within the United States [5].

PK is a well-established and effective method for treating corneal opacities and deformities. However, it carries notable risks, especially owing to its open-sky nature during surgery. This open-sky approach can lead to complications like positive vitreous pressure, observed in approximately 40–50% of cases [6]. Positive vitreous pressure can manifest as a persistently shallow anterior chamber, recurrent iris prolapse, zonular rupture, and complications during cataract extraction such as posterior capsule bulging or rupture, vitreous prolapse, and lens prolapse [7]. Another significant and sight-threatening complication associated with the open-sky nature of PK is suprachoroidal hemorrhage, which occurs with an incidence rate between 0.45 and 1.08% [8].

The survival rates for PK grafts show a variance based on the indication and the nature of the graft. For first-time grafts, the survival rate is approximately 90% at 5 years and 82% at 10 years. However, initial regrafts demonstrate lower survival rates, with only 53% at 5 years and 41% at 10 years. The highest survival rates are noted in primary grafts for keratoconus (97% at 5 years and 92% at 10 years) and Fuchs' dystrophy (97% at 5 years and 90% at 10 years). In contrast, primary grafts for conditions like aphakic bullous keratopathy without intraocular lens placement have significantly lower survival rates, with 70% at 5 years. [9] Additionally, in the literature, the reported complications rates for PK are varied and include microbial keratitis (3%–32%), endothelial rejection (13.1%–26%), endophthalmitis (0.2%–7%), secondary glaucoma (8.1%–30%), graft failure (18%) [10–13], and significant astigmatism (>5 D) in up to 20% of cases [14].

These factors necessitate an enhanced protective approach during transplantation, particularly in cases with heightened surgical risk factors. Such factors include ocular-related issues such as zonular instability, shallow anterior chambers, anesthesia-related complexities, and patient-specific concerns, including advanced age and hypertension.

The development of protective corneal transplantation techniques, dating back to their initial description in 1935, has evolved in response to these risk factors [15–20]. Our study introduces a novel approach—protective penetrating keratoplasty (PPK)—and provides a retrospective analysis of 22 cases. PPK shows particular applicability for conditions such as pseudophakic bullous keratopathy (PBK), especially when stromal scarring is present and in contexts where EK is constrained by the resource limitations typical of developing countries [21]. By evaluating the safety, outcomes, and effects of PPK on corneal endothelial cell density, this investigation seeks to assess its utility as a preferable alternative to conventional penetrating keratoplasty (PK), particularly in complex scenarios. The objective of this analysis is to expand the corpus of corneal transplantation methodologies and lay the groundwork for further detailed investigations, with a keen focus on enhancing applicability in resource-constrained settings.

## 2. Materials and Methods

This retrospective study was conducted at Hospital Hermanos Ameijeiras and included 22 patients (22 eyes; nine males and 13 females) who underwent PPK between February 2018 and December 2021. The inclusion criteria targeted patients with PBK characterized by severe and persistent corneal edema and scarring, unresponsive to conventional medical treatments, and a preoperative corrected distance visual acuity (CDVA) of 0.1 or lower. Exclusion criteria included patients who had previously undergone endothelial keratoplasty, were unable to undergo surgery under general anesthesia, or had undocumented visual acuity.

Within this patient group, all exhibited long-standing, severe corneal edema coupled with significant corneal scarring. Additionally, three of these individuals presented with peripheral anterior synechia affecting one quadrant. Given these conditions and the complex challenges associated with correct alignment and effective treatment of stromal scarring during EK, the decision was made to proceed with PPK.

### 2.1. Surgical Procedure

All surgeries were performed under general anesthesia by a single surgeon. Preoperative preparation included topical ophthalmic drops of gentamicin every 4 hours, initiated 48 hours prior to surgery.

**Figure 1:**
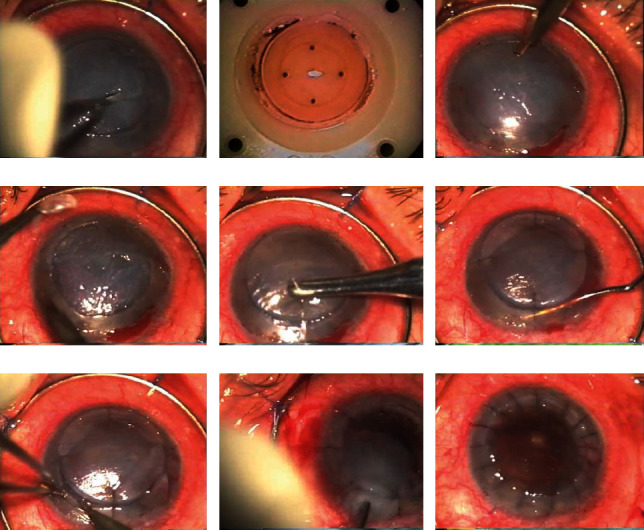
Photographs of the major steps in the protective penetrating keratoplasty procedure. (a) The cornea was marked with a 7.5-mm manual trephine, and the corneal epithelium was removed. (b) The donor cornea was trephinated from its endothelial face using an 8.0-mm Barron trephine on a Teflon block. (c) Nonpenetrating incisions in four quadrants and lamellar stromal dissection were performed. (d) Lamellar dissection was performed in up to 80% of the recipient's stroma. (e) The donor corneal tissue was placed and secured to the recipient's bed. (f) If necessary, synechiotomy was performed in the incised sector in patients with anterior chamber iris synechiae. (g) Continuous anterior chamber penetrating incisions (1/4 circumference each) were made. A suture was placed in the middle of each 1/4 to secure the donor cornea in the incised sector. (h) The remaining recipient corneal tissue was extracted from the unsutured quadrant. (i) Complete interrupted corneal suturing was performed.

**Figure 2:**
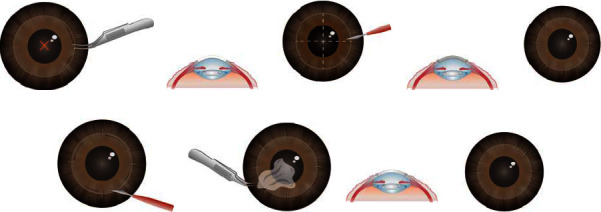
Sketches of the protective penetrating keratoplasty procedure. (a) The cornea was marked with a 7.5-mm manual trephine, and the corneal epithelium was removed. (b) Nonpenetrating incisions in four quadrants and lamellar stromal dissection were performed in up to 80% of the recipient's stroma. (c) The donor corneal tissue was placed and secured to the recipient's bed. (d) Continuous anterior chamber penetrating incisions (1/4 circumference each) were made. A suture was placed in the middle of each 1/4 to secure the donor cornea in the incised sector. (e) The remaining recipient corneal tissue was extracted from the unsutured quadrant. (f) Complete interrupted corneal suturing was performed.

1. A Flieringa Scleral Fixation Ring (Fimco, Perpignan, France) was used to stabilize the surgical field.
2. The host cornea was marked and trephinated to 80% stromal depth using a 7.5-mm manual trephine. Subsequently, the epithelium was removed using Colibri Forceps with tying platforms (Fimco, Perpignan, France) (Figures 1(a) and 2(a)).
3. The donor cornea was trephinated from its endothelial side using an 8.0-mm nonvacuum Barron trephine punch (Tecfen Medical, CA, USA) mounted on a Teflon block (Figure 1(b)). Subsequently, the donor cornea was placed on a Healon GV® viscoelastic bed (Johnson and Johnson, FL, USA) in a Petri dish. This cohesive ophthalmic viscoelastic device (OVD) was crucial in protecting the donor endothelium, creating a protective barrier that minimized the risk of mechanical trauma in subsequent procedural steps.
4. Nonpenetrating incisions were made along the 3-to-9 o'clock and 12-to-6 o'clock axes using a 45° single-edge diamond knife (Duckworth and Kent Ltd., United Kingdom), dividing the cornea into four quadrants. Subsequently, a stromal bed was prepared through deep stromal lamellar dissection, performed quadrant-by-quadrant using the same 45° single-edge diamond knife, and approximately 80% of the corneal stroma was removed (Figures 1(c), 1(d), and 2(b)).
5. The donor corneal tissue was positioned and secured to the stromal bed with 4 nylon 10-0 sutures (Mani, Inc., Tochigi, Japan) at 12, 3, 6, and 9 o'clock positions (Figures 1(e) and 2(c)). The OVD layer continued to serve as a protective interface between the donor and host.
6. A penetrating incision was made into the stromal bed to access the anterior chamber. In cases with anterior chamber iris synechiae, a synechiotomy was performed in the incised sector as necessary (Figure 1(f)). In cases where haptic dislocation was discovered, the intraocular lens (IOL) was carefully rotated to reposition the dislocated haptic into the capsular bag.
7. Initially, the donor cornea was gently lifted in one quadrant using 0.12-mm single tooth forceps (Fimco, Perpignan, France) to facilitate visibility and control. Subsequently, continuous penetrating incisions were made along the groove in the stromal bed within the anterior chamber using a 45° single-edge diamond knife. A nylon 10-0 suture was placed at the midpoint of the incised sector to secure the donor cornea (Figures 1(g) and 2(d)). This procedure was subsequently and symmetrically replicated in the remaining three quadrants.
8. Upon completing the incisions and sutures in all four quadrants, the fully separated stromal bed within the anterior chamber was extracted using delicate forceps—smooth, angled 45°, 9 cm, with tip dimensions of 0.4 × 0.3 mm (Fine Science Tools, Heidelberg, Germany) (Figures 1(h) and 2(e)). The OVD layer aided in minimizing potential endothelial cell loss during the stromal bed extraction.
9. A complete interrupted corneal suture using nylon 10-0 was executed, adhering to the standard PK technique, with all suture knots buried (Figures 1(i) and 2(f)).
10. Intracameral air and antibiotics (cefuroxime [1 mg/mL], 0.2 mL) were added, along with subconjunctival betamethasone (0.1%, 1 mL).

In the surgical procedure of PPK, precision in aligning the donor and recipient tissues is a critical aspect. Initially, sutures are placed at the 12, 3, 6, and 9 o'clock positions directly on the stromal bed, which retains about 20% of its thickness after dissection. This arrangement may initially cause a slight mismatch in alignment. To address this, the initial sutures are slightly tightened more than usual. The inherent elasticity of the corneal stroma allows for a subtle posterior shift of the donor tissue once the residual stromal bed is fully excised, ensuring precise alignment with the recipient's bed. For sutures added before the full excision of the stromal bed, the bed's open state during suturing helps minimize potential alignment issues. After all sutures are in place, any suboptimal alignment between the donor and recipient tissues is corrected by meticulously removing and replacing any problematic sutures, ensuring the precise alignment of the donor cornea with the recipient bed.

The postoperative treatment plan, consistent with the traditional PK procedure, included rigorous follow-ups and specific medication regimens. In the first two months, patients received ciprofloxacin 0.3% (Quimefa, Cuba) one drop every three hours in the first and second weeks and then every six hours from the third week to the end of the second month. Prednisolone 0.5% (Quimefa, Cuba) was administered one drop every three hours in the first week and then every six hours from the second week to two months, adjusted based on patient progress.

Starting from the third month, patients were prescribed gentamicin 0.3% (Quimefa, Cuba) one drop every 12 hours, and prednisolone 0.5% one drop every 12 hours up to six months, both dependent on the patient's clinical evolution. Artificial tears (Quimefa, Cuba) were also administered one drop every 12 hours for up to six months. These regimens were applied uniformly across all patients, as there were no cases requiring special antirejection treatment such as for significant epithelial and endothelial rejection. Regular follow-ups continued weekly for the first two months, then bimonthly until the end of the first year. During consultations, transplant status, transparency, sutures, tonometry, drug tolerance, complications, and treatment were evaluated.

This study measured CDVA in decimal units using a calibrated Snellen chart at a distance of 6 meters. The endothelial cell density (ECD) for donor corneas was evaluated preoperatively utilizing a Konan Eye Bank Kerato Analyzer (Konan Medical Inc., Hyogo, Japan), ensuring that only corneas with sufficient endothelial health were selected for transplantation. Postoperative ECD measurements were conducted at the 12-month mark using a Topcon SP-3000P specular microscope (Topcon Corporation, Tokyo, Japan). Despite the presence of postoperative macular degeneration in three patients, their results were retained in the CDVA analysis to ensure comprehensive outcome reporting.

### 2.2. Statistical Analysis

Data were Figures 1 and 2 obtained from the medical records of patients and processed using Microsoft Excel and SPSS for Windows version 27 (IBM Corp., Armonk, NY, USA). Qualitative variables were expressed as absolute and relative frequencies and/or percentages; quantitative variables were represented as mean, standard deviation, and percentiles, with a 95% confidence interval. Statistical significance was set at *p* ≤0.05.

### 2.3. Ethical Aspects

All patients received a detailed explanation before surgery and signed an informed consent form to ensure an understanding of the research process, potential risks, and potential benefits.

Approval was obtained from the institution's Medical Ethics and Scientific Committee. The study complied with the Principles of Medical Ethics, and patient confidentiality and anonymity were maintained.

## 3. Results

The study included 22 patients (22 eyes; nine males and 13 females) with a mean age of 74.1 ± 8.1 years (range 60–92 years). All 22 surgeries were successful, with one intraoperatively discovered haptic dislocation, and no other complications (IOL removal, explosive choroidal hemorrhage, vitreous prolapse, iris prolapse, or injury) were observed. During the one-year follow-up of the 22 cases, aside from one instance of bacterial keratitis (due to delayed treatment and medication usage due to the coronavirus disease [COVID-19] pandemic, resulting in corneal turbidity and necessitating retransplantation), the grafts remained clear in the remaining 21 cases, without any signs of graft rejection, secondary glaucoma, or suture-related complications.

Table 1 shows the central tendency values for the postoperative visual acuity at 12 months (mean and median), with higher postoperative values. This finding suggested a statistically significant improvement in visual acuity in patients receiving the protective surgical technique (*p* ≤ 0.001), with CDVA reaching or exceeding 0.1 in 17 eyes (77.2%) and reaching or exceeding 0.5 in nine eyes (40.9%).

As shown in Table 2, the ECD at 12 months decreased from an average of 2768 ± 43.91 cells/mm^2^ preoperatively (range 2677–2859 cells/mm^2^) to 2451 ± 64 cells/mm^2^ at 12 months (range 2318–2584 cells/mm^2^), reflecting a loss of 9.2%. One eye was excluded from corneal endothelial cell density analysis because of corneal turbidity caused by bacterial keratitis.

## 4. Discussion

While the rise of EK and DALK has undeniably revolutionized corneal transplantation, particularly in high-resource settings, the broader application of these advanced techniques remains limited in regions where technical and economic barriers prevail. Our study's focus on PPK responds to these global disparities, offering a viable, safer alternative to traditional PK that is more accessible and practical under varied healthcare infrastructures. This is crucial in treating complex corneal conditions, including PBK, where EK may not be the most suitable option owing to associated complications such as severe corneal edema, stromal scarring, stromal haze, and peripheral anterior synechia. The successful implementation of PPK in these cases demonstrates its potential as an adaptable, universally applicable surgical method, especially in environments where the advanced infrastructure required for EK is not available [4].

The success of PPK in the 22 cases detailed in our retrospective study signifies an advancement in reducing perioperative complications and maintaining the clarity of the grafts in 21 cases. This innovative technique offers several advantages, notably preventing sudden intraocular pressure fluctuations, avoiding complete anterior chamber exposure, and minimizing accidental damage to anterior and posterior tissues. A pivotal aspect of our PPK approach is the incorporation of lamellar dissection, strategically employed to create a soft, thin donor bed. This step addresses a key challenge in corneal transplantation: preserving endothelial cells. Direct suturing of the graft onto the trephined host cornea often leads to significant endothelial cell loss owing to friction and pressure [15]. By forming a more compliant donor bed through lamellar dissection, we mitigate this risk, ensuring better preservation of these cells. Additionally, this method allows for the direct removal of the residual donor bed without exposing the anterior chamber. This protects the endothelial layer and streamlines the surgical process.

Compared to traditional PK, the reduced surgical risk of PPK may foster broader adoption. In patients with PBK, particularly those postcataract extraction with IOL implantation, traditional PK presents specific risks. The incidence of complications like positive vitreous pressure in traditional PK is reported to be around 40–50% [6]. These complications can lead to serious issues such as IOL dislocation, haptic rupture, persistently shallow anterior chamber, recurrent iris prolapse, and vitreous prolapse.

In our study, we observed a notable reduction in these complications. No incidents of complications are typically associated with positive vitreous pressure, such as IOL dislocation or haptic rupture. Furthermore, none of our patients required IOL removal owing to IOL dislocation or haptic rupture, which are frequent complications in traditional PK for PBK patients. This indicates that the closed, controlled surgical environment of PPK plays a crucial role in minimizing these risks.

Regarding the potential of PPK in reducing the incidence of severe complications such as suprachoroidal hemorrhage, our approach might contribute to a lower incidence rate than that reported in traditional PK, which ranges between 0.45 and 1.08% [8]. However, it is important to consider the potential confounding factor of the anesthesia protocol, as our approach includes general anesthesia [22]. While our findings are promising, large-scale, prospective studies are necessary to definitively evaluate whether PPK can, indeed, further reduce the incidence of such severe complications.

Our approach contrasts with methods such as Huang et al.'s stepwise decreasing of vitreous pressure by anterior vitrectomy and Cheung et al.'s “Basket” mattress suture [19, 20], avoiding additional intraocular procedures that could increase the risk of postoperative corneal rejection [23]. Additionally, our technique demonstrates potential advantages in limiting endothelial cell loss, a significant concern in corneal transplantation.

While our study primarily focused on patients with PBK, it is worth noting that the technique may have broader applications. The literature suggests that patients with PBK, in particular, stand to gain from avoiding additional surgical procedures like anterior vitrectomy [23]. Moreover, avoiding procedures that could cause iris damage, such as the removal and reimplantation of the IOL, could minimize endothelial cell loss [24]. Therefore, we believe that this surgical modification could be beneficial in various complicated cases. For instance, in pediatric patients, conventional PK is often complicated due to anatomical and physical features such as eyeball crimping, lens and iris displacement, and vitreous surge [25]. The technique is also applicable to vitrectomized patients and those with other surgical risk factors such as hypertension and advanced age.

In our study, PPK demonstrated promising improvements in visual acuity outcomes compared to traditional PK. A higher proportion of eyes in our PPK study achieved a CDVA of 0.1 or more, with a significant percentage reaching 0.5 or higher, suggesting superior visual outcomes with PPK. This improvement is notable when compared to outcomes from traditional PK, where postoperative CDVA improvements have been more modest, typically ranging from 0.01 to 0.16 and averaging around 0.07 one-year postoperation [23, 26, 27]. These comparative insights underscore the potential of PPK as a more effective surgical option for corneal transplantation, particularly in enhancing postoperative visual acuity.

Our study aligns in several key aspects with the work of Iva Dekaris and colleagues, who also aim to advance PK techniques. Both our study and theirs employ protective measures, such as avoiding the complete opening of the entire cornea and using viscoelastic material as a protective barrier [25]. However, our methodology diverges in the incorporation of deep anterior lamellar dissection. This technique has been observed to contribute to a lower rate of endothelial cell loss in our study, making it a potential alternative for cases where endothelial cell preservation is a priority. It is worth noting that the technique employed by Dekaris et al. did not include deep anterior lamellar dissection. This difference in methodology may account for our observed lower rate of endothelial cell loss (9.2%) compared to the 33.2% reported by Dekaris et al. Additionally, the difference in postoperative visual acuity, assessed in decimal units (0.3 in our study vs. 0.7 in theirs), could be attributed to the distinct patient populations each study focused on. While we concentrated on PBK, their study primarily involved keratoconus patients [28].

In contrast, we find it relevant to compare our results with those of Arslan et al., who also aimed to improve PK techniques but arrived at different outcomes [16]. They reported a higher endothelial cell loss of 26.2% at 12 months postoperation. The absence of deep lamellar dissection in their methodology could be a contributing factor. This observation further substantiates our view that the implementation of deep lamellar dissection may be beneficial in reducing endothelial cell loss. Our study yielded an average CDVA of 0.3, assessed in decimal units, with variations in visual outcomes potentially attributable to different patient demographics compared to Arslan et al., who studied a more diverse range of conditions.

Our surgical approach aligns closely with that of Chen et al., particularly in the use of deep lamellar dissection and a viscoelastic material at the donor-host interface [17]. However, our technique minimizes the use of OVD by avoiding its injection into the anterior chamber. This modification likely accounts for our lower endothelial cell loss rate (9.2% vs. Chen's 25.7%). We attribute this advantage to our surgical approach, which avoids the injection of OVD into the anterior chamber. Studies such as Acar et al. indicated that eyes previously subjected to PK have a more fragile corneal endothelium, and Hayashi et al. identified “greater infusion volume” as an independent predictor of endothelial cell loss [29, 30]. In our procedure, only a minimal amount of OVD is present in the anterior chamber. This OVD originates from the Healon GV® viscoelastic bed, serving to protect the donor endothelium. Because of this minimal use, there is no need for the aspiration of OVD to control postoperative intraocular pressure. By avoiding the injection and subsequent aspiration of OVD, we eliminated what could be considered a form of “greater infusion volume,” thereby potentially reducing the risk of endothelial cell loss.

Regarding visual acuity, 40.9% of eyes in our study reached or exceeded a CDVA of 0.5, assessed in decimal units, comparable to the 53.3% reported by Chen et al. These similar outcomes, despite differing patient populations, further underscore the potential efficacy of our surgical approach.

Given the aforementioned comparisons, our technique offers distinct advantages over other methods reported in the literature. Specifically, the use of deep anterior lamellar dissection and the minimized use of OVD contribute to significantly lower rates of endothelial cell loss. These modifications also result in comparable, if not better, visual outcomes. Our findings revealed that our surgical approach may offer a more effective and safer alternative for corneal transplantation, particularly for patients with PBK.

While our technique shows promising advantages in terms of endothelial cell loss and visual outcomes, it is crucial to consider the limitations of this study to appreciate its clinical implications fully.

In addressing the critical issue of endothelial cell loss associated with the suturing of a full thickness cornea onto a dissected host cornea, our study implemented targeted strategies to mitigate this outcome. Specifically, we maximized anterior lamellar excision, removing approximately 80% of the stromal tissue, to reduce the mechanical stress exerted by the cardinal sutures. Additionally, we employed a cohesive OVD to establish a protective barrier between the donor and host corneas, minimizing mechanical stress and friction. Importantly, we strategically avoided the use of OVD within the anterior chamber to prevent the additional stress associated with its removal. These combined measures contributed to a notable reduction in the endothelial cell loss in our study compared to the traditional penetrating keratoplasty method, which exhibits a 27.7% ± 11.1% loss rate after 12 months [31], thus underscoring the efficacy of our approach.

In our research, the concept of a subtle posterior shift of the donor tissue after the complete removal of the residual stromal bed was inspired by Chen et al. findings [17]. While this hypothesis is underpinned by logical deduction and supported by indirect evidence from similar surgical techniques, we must acknowledge the absence of direct, empirical validation of this specific phenomenon within the framework of our protective penetrating keratoplasty (PPK) approach. The nuanced nature of this shift presents significant challenges for direct observation, particularly under standard clinical settings.

While our findings offer promising insights, we must acknowledge the limitations that accompany them. The retrospective nature of this study, coupled with its relatively small sample size, moderates the strength and robustness of our conclusions. Additionally, the study's specific focus on indications for corneal transplantation may limit the generalizability of our results. A significant limitation involves the absence of uniform postoperative corneal topography data. This absence is due not only to the varied timing of suture removal among patients but also to the fact that not all patients had all their sutures removed by the end of the follow-up period. These factors have restricted our ability to comprehensively evaluate the impact of surgery on corneal shape and astigmatism.

In response to these challenges and to address the specific issue of the posterior shift observed with our PPK technique, we commit to methodological improvements in our future research. This includes extending the follow-up period to 24 months to ensure comprehensive suture removal and uniform data collection, coupled with the systematic acquisition of both preoperative and postoperative corneal topography data. Moreover, we plan to employ state-of-the-art imaging techniques and sophisticated quantitative analyses to thoroughly investigate the phenomenon of the posterior shift. This in-depth investigation aims to provide definitive evidence for the occurrence and implications of the posterior shift following the PPK procedure, which is crucial for enhancing the reliability and safety of corneal transplantations. These enhancements are designed to yield a more complete evaluation of the PPK technique's impact on patient recovery and long-term visual function, thereby not only addressing current limitations but also contributing significantly to the safety, efficacy, and advancement of ophthalmic surgical practices.

In our study, one case involved intraoperatively discovered IOL haptic dislocation, initially obscured by corneal edema and likely linked to prior cataract surgery complications. This issue was detected owing to the increased stromal transparency achieved through deep lamellar dissection. Our PPK technique, designed to maintain stable posterior pressure, allowed repositioning of the dislocated IOL, demonstrating the method's adaptability and safety for managing unexpected intraoperative findings.

The PPK technique also features an easier learning curve than traditional surgery, owing to the ease of suturing with a stable anterior chamber. The design of sutures, maintaining a balanced force, effectively reduces the risk of suture-related complications. Our findings confirm this, with no suture complications observed, underscoring the procedure's safety and reliability.

The findings of our study also highlight the necessity of adapting and evolving corneal transplantation techniques to address specific clinical challenges, such as those posed by PBK. Despite the advancements in corneal surgery, the unique demands of certain conditions and patient populations require tailored approaches. Our PPK method, emphasizing reduced endothelial cell loss and enhancing surgical safety, complements the existing array of corneal transplantation techniques and fills a critical gap where these techniques may fall short. This adaptability is particularly significant given the diverse range of corneal pathologies encountered in clinical practice, reaffirming the importance of technique diversification in ophthalmic surgery.

Future research should corroborate these findings through large-scale prospective studies to ensure a more comprehensive understanding of PPK's potential. Specifically, we are currently conducting a controlled study comparing PPK with traditional open-sky PK. The ongoing research aims to evaluate intraoperative complications, including suprachoroidal hemorrhage and positive vitreous pressure, and other outcomes such as visual acuity, corneal endothelial cell loss, and postrejection corneal opacity. This will provide a more comprehensive understanding of PPK's potential and address some of the limitations of the current study. An extended follow-up of these cases would also shed additional light on the long-term efficacy and safety of this approach, particularly regarding corneal endothelial cell loss and graft survival.

In conclusion, this retrospective analysis of 22 patients highlights the potential benefits of PPK in modern clinical practice. Although offering substantial advantages over traditional PK techniques, approaching these findings with caution is crucial, considering the limitations of this study. Further research is needed to validate the applicability and sustainability of these promising results in corneal transplantation.

## Figures and Tables

**Table 1:** Central tendency and dispersion measure values for preoperative and postoperative visual acuity.

Indicators	CDVA		*p*
	Preoperative	Postoperative	
*N*	22	22	
Mean (SD)	0.050 (0.000)	0.341 (0.200)	
95% CI	0.050–0.050	0.252–0.430	
Median	0.050	0.300	≤0.001^*∗*^
IQR	0.000	0.300	

^*∗*^Wilcoxon test for related samples (before and after) for distribution comparison. *N*: count; SD: standard deviation; CI: confidence interval; IQR: interquartile range; CDVA: corrected distance visual acuity.

**Table 2:** Central tendency and dispersion measure values for preoperative and postoperative endothelial cell density.

Indicators	ECD (cellular/mm^2^)		*p*
	Preoperative	Postoperative	
*N*	21	21	
Mean (SD)	2768.05 (43.48)	2451.05 (63.81)	
95% CI	2677.35–2858.75	2317.94–2584.15	
Median	2780	2380	<0.001^*∗*^
IQR	339	482	

^*∗*^Wilcoxon test for related samples (before and after) for distribution comparison. *N*: count; SD: standard deviation; CI: confidence interval; IQR: interquartile range; ECD: endothelial cell density.

## Data Availability

The raw data and analysis files used to support the findings of this study are available from the corresponding author upon request.
